# Autoprobiotics as an Approach for Restoration of Personalised Microbiota

**DOI:** 10.3389/fmicb.2018.01869

**Published:** 2018-09-12

**Authors:** Alexander Suvorov, Alena Karaseva, Marina Kotyleva, Yulia Kondratenko, Nadezhda Lavrenova, Anton Korobeynikov, Petr Kozyrev, Tatiana Kramskaya, Galina Leontieva, Igor Kudryavtsev, Danyang Guo, Alla Lapidus, Elena Ermolenko

**Affiliations:** ^1^Department of Molecular Microbiology, Institute of Experimental Medicine, Saint-Petersburg, Russia; ^2^Department of Fundamental Medicine and Medical Technologies, Saint-Petersburg State University, Saint-Petersburg, Russia; ^3^Institute of Agro-food Science and Technology, SAAS, Shandong, China

**Keywords:** autoprobiotics, microbiota, intestinal dysbiosis, anti-inflammatory, microbiome

## Abstract

Human microbiota is a complex consortium of microorganisms involved in the proper functioning of almost every system of the organism. Majority of the human diseases are associated with the development of intestinal dysbiosis. Dysbiotic condition or dysbiosis is a key pathogenic condition causing many severe infectious or non-infectious diseases. Rapid return to the original microbiota in many cases leads to the fast recovery from the disease. However, the optimal way of the treatment of dysbiosis is still under the discussion. Recently we have developed a method of autoprobiotics based on using isolated indigenous bacteria for improving of microbiota condition. The method based on feeding the patients with bacterial products grown from their personal, genetically characterised strains have been successfully tested in clinic on patients with IBS or chronic pneumonia. In present study we tried to evaluate technology employing autoprobiotic bacteria belonging to different species employing the rat model of antibiotic induced dysbiosis. Six experimental groups of animals after taking antibiotics were treated with different variants of autoprobiotics (lactobacillus, bifidobacteria, enterococcus, their mixture, fecal microbiota, or anaerobically grown complex of indigenous microbiota) prepared for each of them before the development of dysbiosis. Judging by the multiple parameters including metagenomics analysis of microbiota, immune status and microbiota content of the animals with dysbiosis relatively to control group, the most pronounced positive changes were provided by autoprobiotics based on enterococci, bifidobacteria or the consortium of indigenous bacteria grown under anaerobic conditions. These groups of autoprobiotics were delivering the most effective restoration of the original microbiota content and significant anti-inflammatory reaction of the immune system.

## Introduction

Human microbiota is a complex consortium of numerous species of bacteria, archaea, viruses, fungi, and protozoa (more than 500 bacterial species in the gut alone). It is now considered as an additional organ in the human body (Suvorov, 2013). The microbiota is an essential factor influencing almost every function of the organism in health and disease. The application of next-generation sequencing (NGS) technology has increased the interest in the role of the microbiota in health and disease. This interest grew rapidly with the advent of several multinational scientific initiatives such as the NIH Human Microbiome Project (https://hmpdacc.org) and MetaHit Consortium (http://www.metahit.eu). Complete genome sequencing of hundreds of bacterial genomes from the human microbiota and development of new microbiome bioinformatics analyses allowed making several significant scientific discoveries. It was determined that the microbiota is tissue-specific and at the same time possesses features which are typical to each individual (Qin et al., 2010). Gut microbiota being the most abundant microbial community in the organism is highly personal but generally favour a limited number of compositions known as enterotypes (Arumugam et al., 2011).

Another significant finding is related to the establishment of the innate immune system. Recently it was determined that gut microbial composition undergoes the most significant changes during the first 3 years of life. This allows establishing immune tolerance to the indigenous microbiota and differentiation between the autochthonous (personal) microbiota from the allochthonous (foreign) bacteria, fungi, and viruses (Wopereis et al., 2014). These discoveries allowed a better understanding of the roots of numerous diseases associated with dysbiosis, including inflammatory bowel disease, multiple sclerosis, diabetes (types 1 and 2), atopy, asthma, autism, and cancer (Lloyd-Price et al., 2016). In most cases, treatment outcome of many infectious or non-infectious diseases is associated with the restoration of the microbiota composition to the initial healthy state. This fact has influenced numerous studies on the effects of bacterial therapy including probiotics or fecal microbiota transplantation (FMT) where microbiota is transplanted from a healthy donor (Vuotto et al., 2014; Gupta et al., 2016). A major limitation of these approaches is that exogenously obtained microbes may be foreign to the recipient's immune system. Another approach for the treatment of dysbiosis is based on autoprobiotics. Autoprobiotics rely on a different strategy of microbial therapy. It involves *in vitro* culture of personal microbiota strains and the preparation of a personalised food or drug for use in microbiome restoration (Suvorov, 2013). The general aim of the present work is to evaluate the therapeutic potential of different bacterial variants of autoprobiotics, employing an experimental model of antibiotic-induced dysbiosis followed by metagenome and immunological analysis.

## Materials and methods

### Animals

Male Wistar rats (200–250 g, 6–7 weeks of age) were obtained from Animals Breeding Center, Rappolovo, Russia. Rats were maintained in separate cages under constant conditions at room temperature (18–22°C), on a 12 h light/dark cycle, with the noise level not exceeding 85 dB, at 50–60% humidity and were provided with free access to water and standard rat pellets (complete compound feeds for laboratory rats and mice, PK-120 sh. 1492, state industry standard R 50258-92 in pellets with diameter 14 mm, Russia). This study was carried out in strict accordance with the necessary ethical requirements and in compliance with the principles of humanity (of the European Communities No 86 / 609 EU). The study was approved by the local ethics committee of FSBSI “IEM.”

### Experiment design

The rats were divided into six experimental and two control groups with 8 animals in each group. All experimental-(LB, BI, EN, MIX, AN, and FE) and control group designated 3AB were treated with antibiotics for 3 days according to the protocol previously published (Ermolenko et al., 2013). Some animals from group 3AB were monitored for an additional 5 days after the fecal microbiota were collected. These animals were grouped as 8AB. After receiving antibiotics for 3 days, rats from the six experimental groups were fed with different preparations of autoprobiotics—lactobacilli, bifidobacteria, enterococci, a mixture of three strains, anaerobically grown fecal microbiota, or indigenous feces previously prepared from their own fecal samples. The second control group (C) received water instead of antibiotics and PBS instead of autoprobiotics (Table 1).

**Table 1:** Experimental design.

**Experiment groups**	**Treatment (1–3 days)**	**Treatment (4–8 days)**	**Samples harvested**
C	Distilled water	PBS	Fecal samples -for microbiota study. On day 9-blood samples and spleen biopsies for immunological analysis.
3AB	Ampicillin + Metronidazole	–	Fecal samples-for microbiota study.
8AB	Ampicillin + Metronidazole	PBS	Fecal samples-for microbiota study. On day 9-blood samples and spleen biopsies for immunological analysis.
BI	Ampicillin + Metronidazole	*Bifidobacterium* spp.	
EN	Ampicillin + Metronidazole	*Enterococcus faecium*	
LB	Ampicillin + Metronidazole	*Lactobacillus* spp.	
MIX	Ampicillin + Metronidazole	Mixture of *Bifidobacterium* spp., *Lactobacillus* spp. and *Enterococcus faecium strains*	
AN	Ampicillin + Metronidazole	Anaerobic consortium	
FE	Ampicillin + Metronidazole	Feces	

### Autoprobiotic strains

Indigenous enterococci, lactobacilli, or bifidobacteria were isolated from feces of rats before the animals were treated with antibiotics and were grown separately. Analyses of stool samples and identification of individual clones of bacteria (*Enterococcus* spp., *Lactobacillus* spp. or *Bifidobacterium* spp.) were performed using selective media or by PCR.

Genetic characterization of the LAB strains (enterococci or lactobacilli) used in the study was carried out using quantitative PCR (qPCR) with species-specific primers and primers for the identification of the virulence-related genes (Table S1). *Enterococcus faecium* was the only enterococcal autoprobiotics selected by genetic analysis. The enterococci were tested for the presence of virulence-related genes (Table S1) and strains carrying these markers were excluded from the study. Indigenous strains of lactobacilli, bifidobacteria, and enterococci were included into the appropriate group of autoprobiotics (LB, BI, and EN) and grown in MRS broth (HiMedia, India), Blaurock broth (Nutrient medium, Russia), and Brain Heart Infusion Broth (Gibco diagnostics, USA), respectively. They were then harvested by centrifugation at 3,000 *g* for 10 m, washed three times with sterile phosphate buffer saline (PBS-8.00 g/l NaCl, 0.20 g/l KCl, 1.44 g/l Na_2_HPO_4_, 0.24 g/l KH_2_PO_4_, pH 7.4, pH 7.2), suspended in sterile PBS with sucrose (20%) and gelatin (10%) at a concentration of 5.5 × 10^8^ CFU/ ml and stored at −20°C until use.

Group MIX received a mixture of selected indigenous enterococci, lactobacilli, and bifidobacteria in equal amounts at a concentration of 5.5 × 10^8^ CFU/ ml.

Bacteria from AN group were prepared by anaerobically growing the complex community of fecal bacteria originally taken from each individual rat and fed to the same animal. For this purpose, feces collected from rats were diluted in PBS and cultivated anaerobically in thioglycollate broth (HiMedia, India) with 10% of sterile fecal extract for 72 h.

Group FE received their own feces diluted in PBS. (Experimental groups are listed in Table 1).

### Microbiota study and immunological analysis

Fecal samples were collected on day 4 and day 9 and were analysed by qPCR and 16S rRNA gene-based metagenomic analysis. Blood samples and spleen biopsies were taken on day 9 for immunological studies.

DNA from feces was isolated using QIAamp DNA Stool Mini Kit (Qiagen) following the manufacturer's protocol. Samples were incubated in the lysis buffer at 90 °C for 10 m for optimal bacterial lysis. For microbiome sequencing, DNA libraries were prepared using the Illumina Nextera sample preparation kit with DNA primers corresponding to V3–V4 regions of the 16S rRNA gene listed in Table S1. Illumina MiSeq was used for sequencing the libraries. Sequencing was performed in Saint-Petersburg University Resource Center ≪ Biobank ≫.

### OTU generation

Fastqc (http://www.bioinformatics.babraham.ac.uk/projects/fastqc) was used to evaluate the quality of raw reads. CD-HIT-OTU-Miseq (Li and Chang, 2017) was used for OTU retrieval. CD-HIT-OTU-Miseq allows retrieving OTU from paired-end reads without merging paired sequences. This was achieved by matching of clustering results for R1 and R2 reads. CD-HIT-OTU-Miseq can use only high-quality regions of reads for clustering. Clustering was performed using the following parameters - lengths of high-quality regions of R1 and R2 read of 200 and 180 bp respectively, 97% read similarity for clustering cutoff and 0.00001 for abundance cutoff. OTUs were annotated using Greengenes database version 13.5 (DeSantis et al., 2006).

### Analysis of microbiome diversity

OTUs present in less than 5% of samples were discarded for noise filtering. R package phyloseq (McMurdie and Holmes, 2013) was used to plot phylum abundances. R function prcomp() was used for principal component analysis (PCA). Vectors for PCA corresponded to OTU abundances filtered for noise and normalised into relative abundances. Noise filtering cutoff was increased for PCA to discard OTUs present in less than 25% of samples. This was done to increase the percent of variance explained by 1st and 2nd principal components and to receive a more representative 2D picture.

qPCR analysis to detect bacteria in the colon was performed using Colonoflor kit (ExPlana, Russia) on the RT-PCR unit Mini-Opticon, BioRad. RT-qPCR data on certain bacterial species were confirmed by classical bacteriology study.

### Determination of cytokines in blood serum

Levels of MCP-1, IL-10, and TGF-β in blood serum was evaluated using ELISA with Bender MedSystems kits (eBioscience, USA) according to the manufacturer's instructions. All samples were tested in duplicates and read on iMark, BioRad, USA.

### Flow cytometry

The phenotype of blood and spleen cells were determined on day 9 by flow cytometry analyses following standard antibody staining procedures. Cells were stained with the combination of following antibodies: CD3, CD4, CD8, CD161, CD25, FOXP3, and CD45RA. Cells were stained with three different combinations of fluorescent-labelled DNA monoclonal antibodies. All antibodies were obtained from BioLegend and used according to the instructions from the manufacturer. The first two combinations of antibodies were used for cell-surface staining, the third one for cell-surface and intracellular staining.

The first combination consisted of FITC-conjugated mouse anti-rat CD3 (clone 1F4), PE-conjugated mouse anti-rat CD8a (clone OX-8), PE/Cy7-conjugated mouse anti-rat anti-CD4 (W3/25 clone), and APC-conjugated mouse anti-rat CD161 (clone 3.2.3, that recognised a common epitope of NKR-1P1a (CD161a) and NKR-P1b (CD161b). The second combination consisted of FITC-conjugated mouse anti-rat anti-CD3 and PE-conjugated mouse anti-CD45RA rat (clone OX-33). Mixtures of antibodies were added to 50 μL of K3-EDTA anticoagulated whole rat blood, gently mixed and incubated for 15 m at room temperature in the dark. Then 250 μL of OptiLyse C Lysing Solution (Beckman Coulter) was added, mixed, and incubated for 10 m at room temperature in the dark. Following the incubation, 250 μL of PBS was added. After 10 m, the samples were washed two times with washing buffer (PBS containing 2% FCS (Sigma Aldrich), and analysed by flow cytometry.

The third combination consisted of two antibodies for cell-surface staining—FITC—conjugated mouse anti-rat anti-CD25 (clone OX-33) and PE-Cy7/conjugated mouse anti-rat CD4 (W3/25 clone), as well as PE-conjugated mouse anti-mouse/rat/human FOXP3 (150 d clone) for intracellular staining. Intracellular staining was performed using the FOXP3 Fix/Perm buffer set (BioLegend). All samples were analysed using a Navios™ (Beckman Coulter) flow cytometer equipped with two diode lasers (488 and 635 nm). Data were analysed using Navios Software v.1.2 and Kaluza™ v.1.2 (Beckman Coulter) software.

## Statistical analysis

Statistical analysis was performed using the software package Statistica 8.0. (StatSoft, USA). Differences between the groups were compared using ANOVA and Kruskal–Wallis tests. *P* ≤ 0.05 was considered significant.

## Results

### Metagenome analysis

In order to characterize microbiome composition, reads were clustered with a threshold of 97%. The microbial species corresponding to the 16S rRNA sequence clusters were identified based on results from previous studies (Li and Chang, 2017). Simultaneous clustering of reads of all analysed samples yielded 7782 OTUs. Most of the OTUs were present in both low sample numbers and low absolute amounts, which is a common feature of gut microbiome samples (Quince et al., 2009). OTUs present in less than 5% of samples were discarded for noise filtering. Most of the low abundant OTUs failed to match any sequences from Greengenes database 13.5. A number of annotated OTUs was 534 with 22.4% unclassified reads.

**Figure 1:**
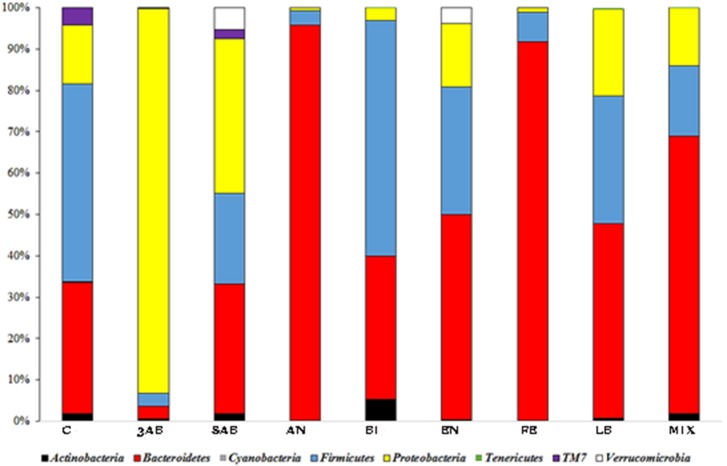
Taxonomic distribution (phylum level) of gut microbiota in different groups of rats. OTU counts corresponding to different groups were summarised, and the relative proportions of detected phyla were plotted.

It was noticed, that all the groups except 3ab, which was comprised predominantly of proteobacteria, were characterised by a significant degree of bacterial heterogeneity. This demonstrated a positive effect of autoprobiotics on the microbiome making it similar to control group C (Figure 1). The high proteobacterial count in group 3AB was due to the effects of the antibiotic cocktail. Bacterial composition of group 8AB, which received only PBS without autoprobiotics for 5 days after antibiotics, became more diverse compared to 3AB, but still remained rich in proteobacteria. Summarised and statistically evaluated results of metagenome data analysis at the phylum level are presented in Table 2. Similar results were obtained after the metagenome analysis at class or family level (Figures S1, S2, and Tables S2, S3).

**Table 2:** Results of comparison of intestinal microbiota member in the experimental groups compared to control group C (Phylum level).

**Phylum/Groups**	**3AB**	**8AB**	**AN**	**FE**	**EN**	**LB**	**BI**	**MIX**
*Actinobacteria*							↑*p* = 0.0015	
*Bacteroidetes*	↑*p* = 0.0005		↑*p* = 0.0003	↑*p* = 0.0005	↑*p* = 0.056			↑*p* = 0.052
*Firmicutes*	↓*p* = 0.0005		↓*p* = 0.0068	↓*p* = 0.0002				
*Proteobacteria*	↑*p* = 0.0005	↑*p* = 0.0001			↑*p* = 0.0147			↑*p* = 0.037

*Arrows represent the direction of relative change in microbiota composition*.

*Grey cells represent table samples with no significant difference from control*.

**Figure 2:**
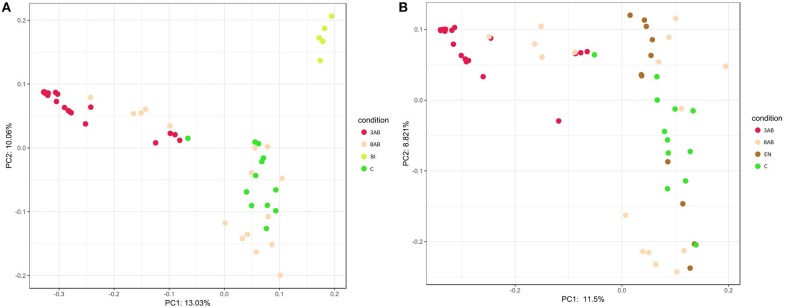
Principal Component Analysis (PCA) of the two groups. **(A)** PCA representation of control groups and group BI. **(B)** PCA representation of control groups and group EN.

A similar result was obtained after PCA of sample OTU compositions. Metagenome compositions of animal groups having bifidobacterial (Figure 2A) or enterococcal (Figure 2B) autoprobiotics colocalised with microbiota of control group C. On the contrary, metagenomes of groups 8AB and 3AB clustered in the opposite part of the graphs.

Analysis of metagenome data revealed significant variation in microbiome composition in “normal” state of control animals, which could be seen by the broad distribution of dots in PCA. Points representing the microbiome after antibiotic-treatments (3AB) formed a more compact group than the points representing the normal microbiome states (control group that received normal saline for all 8 days of the experiment). This illustrated that normal microbiome of animals without antibiotic-treatment was more diverse than the microbiome of animals after antibiotic-induced dysbiosis. “Normal” and “dysbiosis” groups were the most distant, and all autoprobiotics shifted microbiome condition from “dysbiosis” closer towards the control group. The natural recovery group (8AB) also moved towards microbiome restoration, though less evident compared to the autoprobiotics groups. PCA representing microbiomes obtained from groups having other autoprobiotics also followed the same trend. Results obtained employing other types of autoprobiotics were similar but less evident (Figure S3).

**Figure 3:**
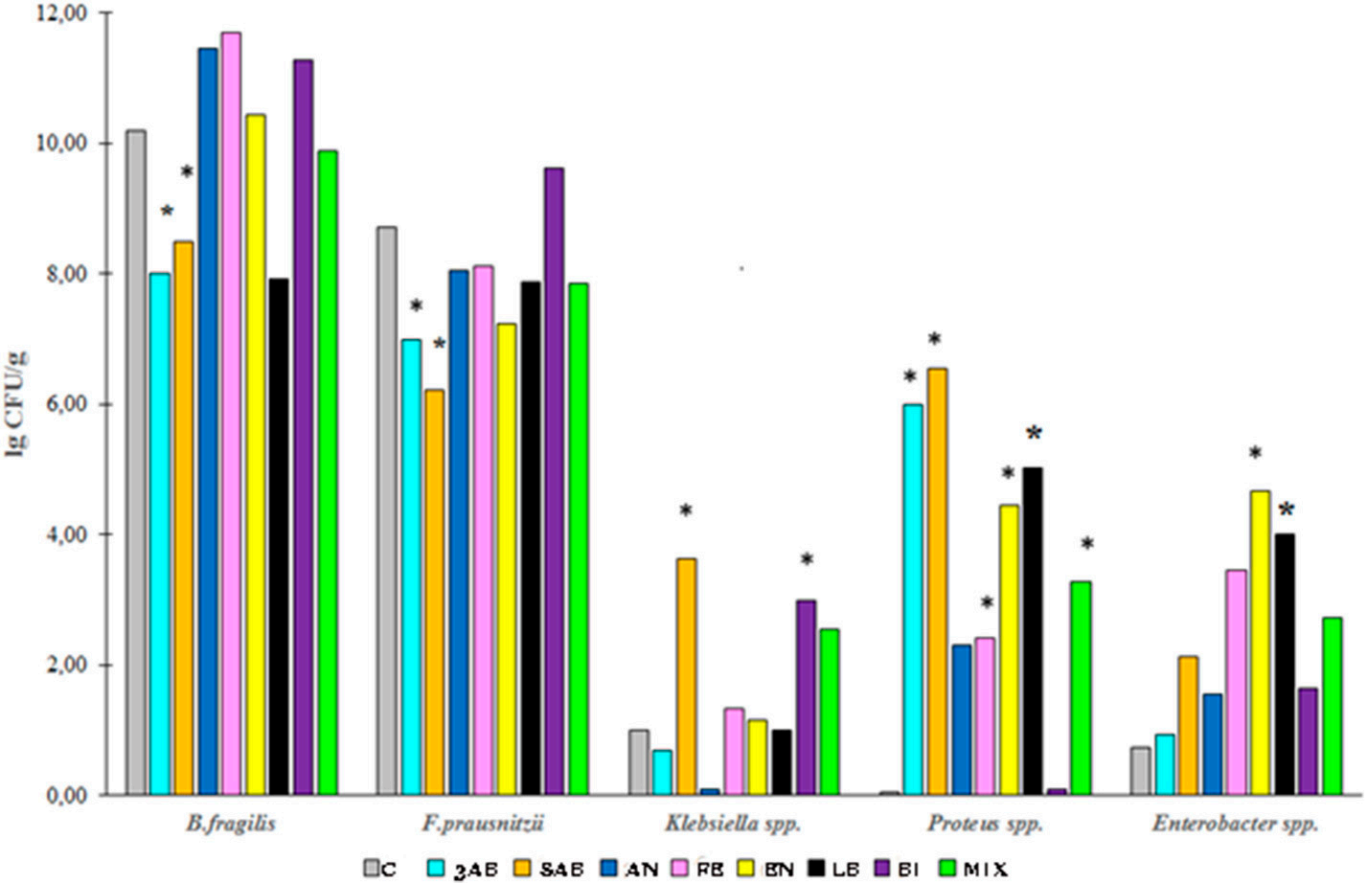
Bacterial content in the fecal samples of rats from different groups. The population of various bacteria in the experimental groups relative to control group C. ^*^p < 0.05.

qPCR analysis of microbiota revealed that antibiotic-treatment selectively depleted most of the marker bacteria in the gut. We also monitored the substantial growth of Gram-negative opportunistic bacteria including *Proteus vulgaris, Klebsiella, Enterobacter*, and *E. coli* (Figure 3). Administration of autoprobiotics triggered a significant degree of microbiota restoration. However, complete restitution was not achieved in any group. Among the autoprobiotics used, auto-bifidobacteria (BI) and the anaerobic mixture (AN) provided the highest increase in *F. prausnitzii* content (Figure 3). Total results of the qPCR analysis are summarised in Table 3.

**Table 3:** Bacterial content in different experimental groups compared to control group C.

**Bacteria/Groups**	**3AB**	**8AB**	**AN**	**FE**	**EN**	**LB**	**BI**	**MIX**
*Enterococcus* spp.	↓*p* = 0.001							
*F. prausnitzii*	↓*p* = 0.0003	↓*p* = 0.04						
*B. fragilis*	↓*p* = 0.001	↓*p* = 0.02						
*Lactobacillus* spp.					↓*p* = 0.001			
*Klebsiella* spp.		↑*p* = 0.022					↑*p* = 0.04	
*Enterobacter* spp.					↑*p* = 0.004	↑*p* = 0.017		
*Proteus* spp.	↑*p* = 0.001	↑*p* = 0.009		↑*p* = 0.016	↑*p* = 0.02	↑*p* = 0.02		↑*p* = 0.03
*E.coli*		↑*p* = 0.050	↑*p* = 0.002	↑*p* = 0.001	↑*p* = 0.011	↑*p* = 0.021	↑*p* = 0.001	↑*p* = 0.007

Grey cells represent the samples with no significant difference from control–p > 0.05.

*Results obtained by Qpcr*.

### Immunological analysis

**Figure 4:**
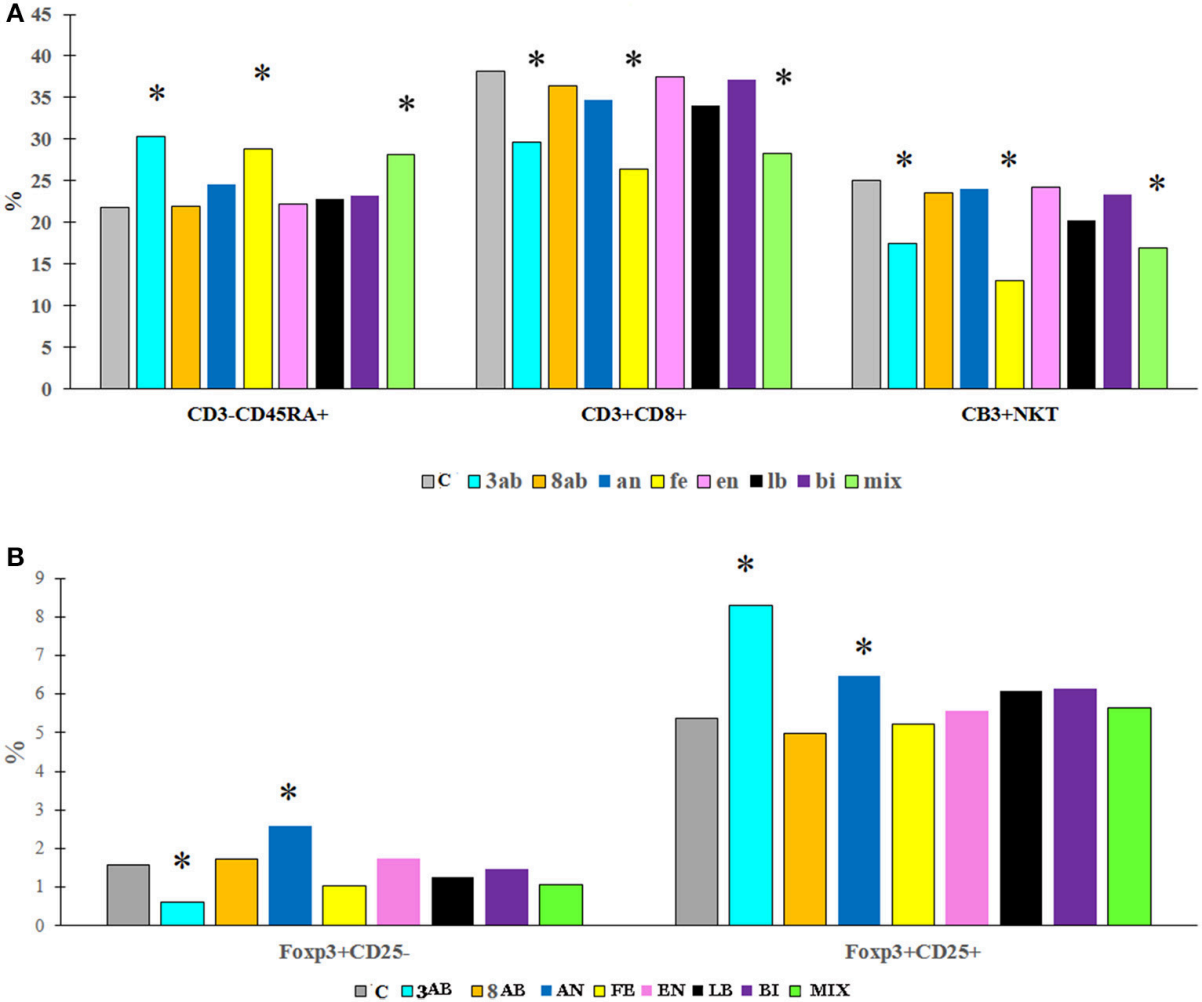
The content (%) of different immune cells in the blood **(A)** and spleen **(B)** of rats from experimental groups and control. ^*^P < 0.05.

Flow cytometry of blood and spleen cells revealed substantial differences between the groups. Antibiotic-treatment induced immunological changes typical of gut inflammation-depletion of the NKT cells in the blood and an increase of the CD4^+^ T cells. We were also able to determine a significant increase in the number of B cells in the blood serum (Figure 4, Table 4).

**Table 4:** Comparison of immunological markers in the study groups.

**Source/Method**	**Parameters/Group**	**3AB**	**8AB**	**AN**	**FE**	**EN**	**LB**	**BI**	**MIX**
Spleen/flow cytometry	CD3^+^CD4^+^	ND	↑*p* = 0.01	↑*p* = 0.03		↑*p* = 0.02			
	CD3^+^CD4^+^CD25^−^ Foxp3^+^	ND		↑ 0.021					
	CD3^+^CD4^+^CD25^+^ FoxP3^+^	ND		↑*p* = 0.039					
Blood/flow cytometry	CD3^+^CD4^+^				↑*p* = 0.03				
	CD3^+^CD8a^+^				↓*p* = 0.004				↓*p* = 0.03
	CD3^+^NKT	↓*p* = 0.008			↓*p* = 0.003				↓*p* = 0.04
	CD3^−^NK								↑*p* = 0.02
	CD3^−^CD45RA^+^ (B cells)	↑*p* = 0.01			↑*p* = 0.004				↑*p* = 0.01
Serum/ELISA	IL-10	ND		↑ 0.04		↑*p* = 0.001	↑*p* = 0.001	↑*p* = 0.03	
	MCP-1	ND							
	TGF-b	ND							

*ND, no data; Grey cells, p > 0.05*.

**Figure 5:**
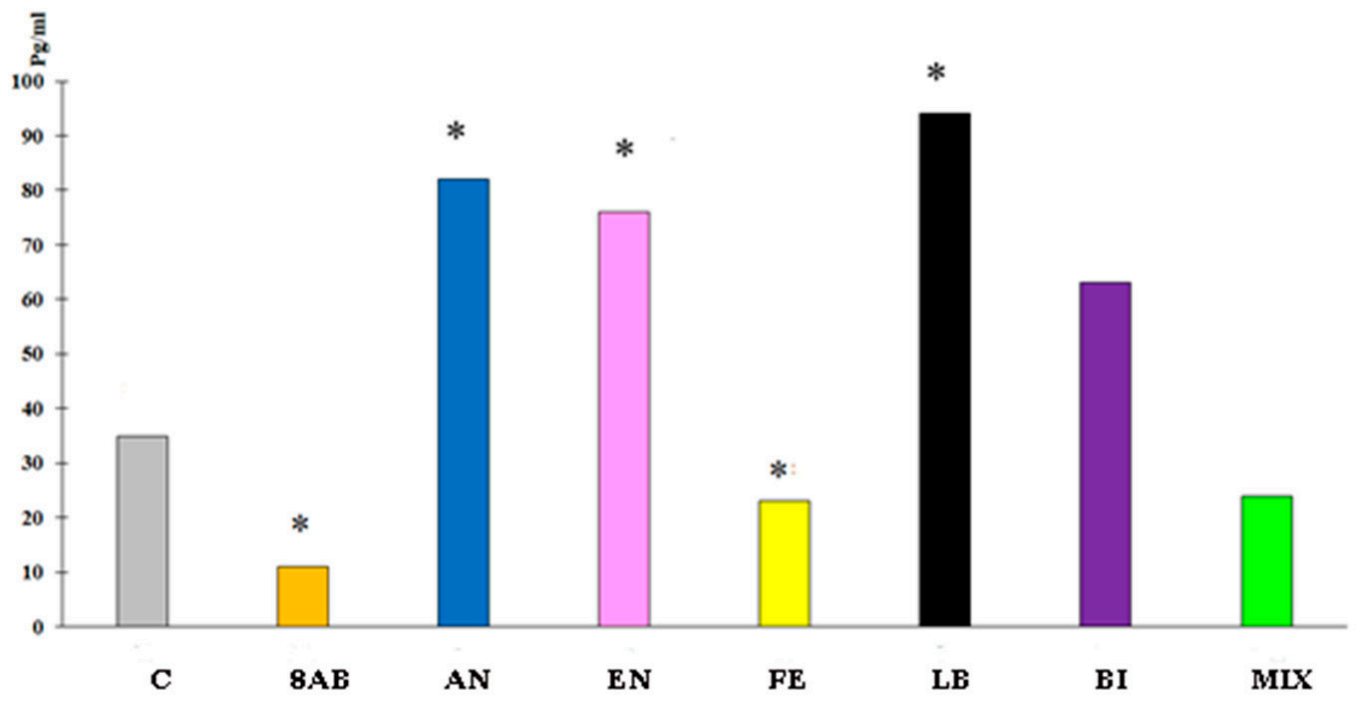
Blood serum IL-10 concentration in the experimental groups compared to control group C. ^*^p < 0.05.

ELISA for cytokines in the blood revealed a decrease in IL-10 and MCP-1 production and slight stimulation of TGF-b in group 8AB. In groups receiving autoprobiotics EN, LB, AN, and BI, we observed an increase in production of IL-10 and MCP-1 in FOXP3^+^CD25^+^ cells. Immunological changes in FE and MIX showed a trend similar to group 8AB (Figure 5, Figures S4A,B, Table 4).

## Discussion

It is well established that human health depends on the condition of the microbiota and that numerous diseases are associated with dysbiosis. It is also well known that various antibiotics or antibiotic cocktails can significantly decrease bacterial abundance and change the microbiota composition (Morgun et al., 2015).

However, in spite of many experimental studies involving antibiotics as a major cause of dysbiosis, very little is known about effective strategies for restoring the microbiota. Bacterial therapy employing probiotics or fecal transplantation have an apparent weakness due to the alien nature of microorganisms introduced into the host. Russian microbiologist Boris Shenderov pioneered the use of indigenous bacteria for the treatment of dysbiosis. He developed and patented an approach based on personal lactobacilli and bifidobacteria for the restoration of microbiota (Shenderov and Manvelova, 1999).

Recently we demonstrated another strategy for dysbiosis treatment, using genetically-tested-patient-derived bacteria grown in laboratory conditions and provided to the patients in the form of fermented milk products. Individually selected and cultured probiotic strains of lactobacillus or enterococcus were successfully tested in the clinic on patients with irritable bowel syndrome (IBS) and pneumonia (Suvorov, 2013). Autoprobiotic milk fermented bacterial products based on enterococci or lactobacilli were able to restore the microbiota in different clinical conditions including irritable bowel diseases (IBD), and ulcerative colitis (UC) (Suvorov et al., 2011; Soloviova et al., 2017). This treatment was also effective in the restoration of microbiota in astronauts (Il'in et al., 2013). No side effects were observed in this study.

In the present study, we addressed a question regarding the selection of the optimal autoprobiotic strain or the best autoprobiotic bacterial composition. In the experiments performed on a rat model of dysbiosis, we tested different bacterial members grown from one selected clone of indigenous bifidobacteria, lactobacilli or enterococcus isolated from feces of the same animal. Together with mono-strain autoprobiotics, we tested three variants of bacterial compositions (fecal biomass, anaerobically grown individual microbiota and the mixture of three different strains of autoprobiotics). Administration of antibiotics (ampicillin and metronidazole) caused depletion in the abundance and richness of microbiota. In 90% of experimental rats, the intestine was dominated by the gamma proteobacteria.

Natural recovery from antibiotics with only normal saline treatment for 5 days (group 8AB) showed positive dynamics, though the microbiome composition did not change as substantially as in autoprobiotics-treated groups. Microbiome analysis of feces obtained from the animals without autoprobiotic treatment revealed a significant degree of variability of the microbiome. However, it was always far apart from the control animals that did not receive the antibiotics. Interestingly, the bacterial composition in the microbiome of the autoprobiotics-treated groups was similar irrespective of the autoprobiotics administered.

Data obtained by qPCR revealed a significant increase in the number of proteobacteria such as *Proteus* spp., *Klebsiella* spp., *E. coli*, and *Enterobacter* spp. together with a dramatic decrease in the amounts of *F. prausnitzii, Enterococcus* spp., and *Bacteroides* spp. after antibiotic-treatments (Table 3). These negative trends in microbiota composition usually reflect inflammation in the gut. Autoprobiotic-administration abolished the inflammation. Different kinds of autoprobiotics changed the bacterial content of microbiota depending on the individual features of each animal and the type of the autoprobiotic preparation used. However, it was possible to determine different trends among the groups. For example, autoprobiotics based on bifidobacteria or anaerobically grown microbiota were able to diminish the amount of Proteobacteria represented by *Proteus* spp. At the same time, autoprobiotic bifidobacteria possessed very low antagonistic activity against *Klebsiella* spp. unlike the other autoprobiotics such as indigenous enterococci.

Comparison of the microbiota revealed that treatment with multi-strain preparations did not have an advantage over mono-strain autoprobiotics. This was in line with the previous study using reparations based on auto-enterococcal strains in the clinic. Microbiota composition in animals treated with indigenous enterococci and bifidobacteria preparations was the closest to the control microbiota. Another group which provided fast recovery was group AN, which received a mixture of bacteria grown in an anaerobic condition.

Immunological changes in the blood of the antibiotic-treated animals were characterised by the proinflammatory trends typical to dysbiotic conditions. All autoprobiotics were able to improve the immunological parameters to a certain extent. But there were significant differences between the groups. The immunological study focused on the major immunologically established landmarks representing the status of gut mucosal inflammation - T cells markers such as FoxP3^+^ CD 25 in spleen and blood, B, NKT cells and regulatory cytokine IL-10 content in blood. These immunological parameters are known to undergo significant changes both in clinical cases of gut inflammatory diseases and in experimental models of antibiotic-induced inflammation (Olszak et al., 2014; Zeissig and Blumberg, 2014). These immunological effects after consuming antibiotics correlated with the decrease in the presence of the major butyrate producer *F. prausnitzii*, which might reflect the decrease in the amount of short chain fatty acids produced in the gut.

Interestingly, distinct immunological changes were observed in autoprobiotics groups. Groups FE and MIX were characterised by the highest increase in B cells and a decrease in NKT cells in blood similar to the group without any autoprobiotics – 8AB. This kind of immunological reaction is typical for the adaptive immune system following inflammation.

On the contrary, mono-strain autoprobiotics from groups BI, LB, and EN together with group AN were characterised by a significant increase in the regulatory cytokine IL-10 and CD 25^+^FoxP3^+^ T cells, reflecting a distinctive anti-inflammatory trend. In this respect, the use of the anaerobically grown indigenous bacteria as probiotics looks very promising (El Enshasy et al., 2016). Apparently, different autoprobiotic preparations can be beneficial in different pathological conditions associated with different variants of dysbiosis. However, further studies are needed for completely understanding the effects of autoprobiotics in controlling dysbiosis.

## Conclusion

An autoprobiotic approach based on the use of personal bacterial strains was evaluated for the restoration of microbiota in a model of antibiotic-associated dysbiosis. Treatment with different autoprobiotic strains led to rapid recovery of microbiota compared to animals with no autoprobiotic treatment. Metagenomic and qPCR data reflected significant changes in microbiota composition and bacterial abundance after taking autoprobiotics. However, complete restoration of the microbiota was not achieved. Immunological changes following autoprobiotic treatment were characterised by a general trend towards the recovery of the immune status. Distinctive anti-inflammatory reactions were observed with administration of autoprobiotics based on bifidobacteria, enterococci or a mixture of bacteria. Anaerobically grown fecal microbiota provided the most promising probiotic effect.

## Author contributions

AS supervised the project and wrote the paper. AK prepared metagenome DNA and performed the preliminary DNA analysis. MK involved in all the stages of work with experimental animals and collected the samples for metagenome sequencing. YK did PCA analysis and helped in editing the paper. NL took the blood samples from the animals and collected fecal microbiota. AK helped in making bio information analysis and DNA primer design. PK did significant part of bio information analysis of fecal microbiota. TK collected the blood from the experimental animals and prepared the samples for ELISA and immune fluorimetry analysis. GL did all immunological studies and ELISA experiments. IK did all immuno fluorimetry studies. DG participated in revising of current publication, critically for important intellectual content, and interpretation of data for the work. AL supervised all bio information work and participated in revising of the manuscript. EE supervised all the experiments, prepared most of figures and tables, and personally worked with experimental animals.

### Conflict of interest statement

The authors declare that the research was conducted in the absence of any commercial or financial relationships that could be construed as a potential conflict of interest.
