# Supplementary material for: Autoprobiotics as an Approach for Restoration of Personalised Microbiota
Source: Front Microbiol. 2018 Sep 12;9:1869. doi: 10.3389/fmicb.2018.01869 (PMC6144954; doi:10.3389/fmicb.2018.01869)
Supplement: Supplementary file 1 [file Data_Sheet_1.docx]

1. **Supplementary Material**

**Figure S1**

Taxonomic distribution (family level) of gut microbiota in different groups of rats under study. OTU corresponding to different groups is summarized*.*


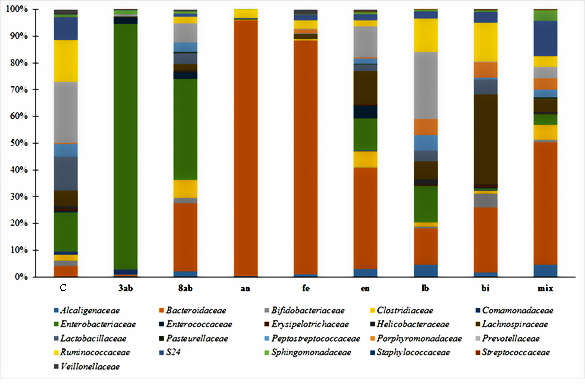


**Figure S2**

Taxonomic distribution (genus level) of gut microbiota in different groups of rats under study. OTU corresponding to different groups is summarized.


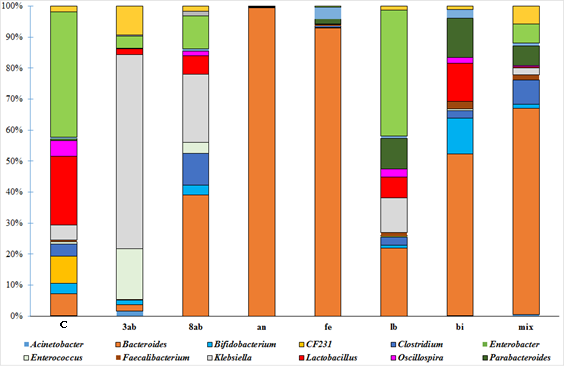


**Figure S3**

PCA representation of control groups and group that received autoprobiotic (A) lactobacillus, (B) mixture of 3 bacterial strains, (C) fecal microbiota, and (D) anaerobically grown bacteria.

A

**
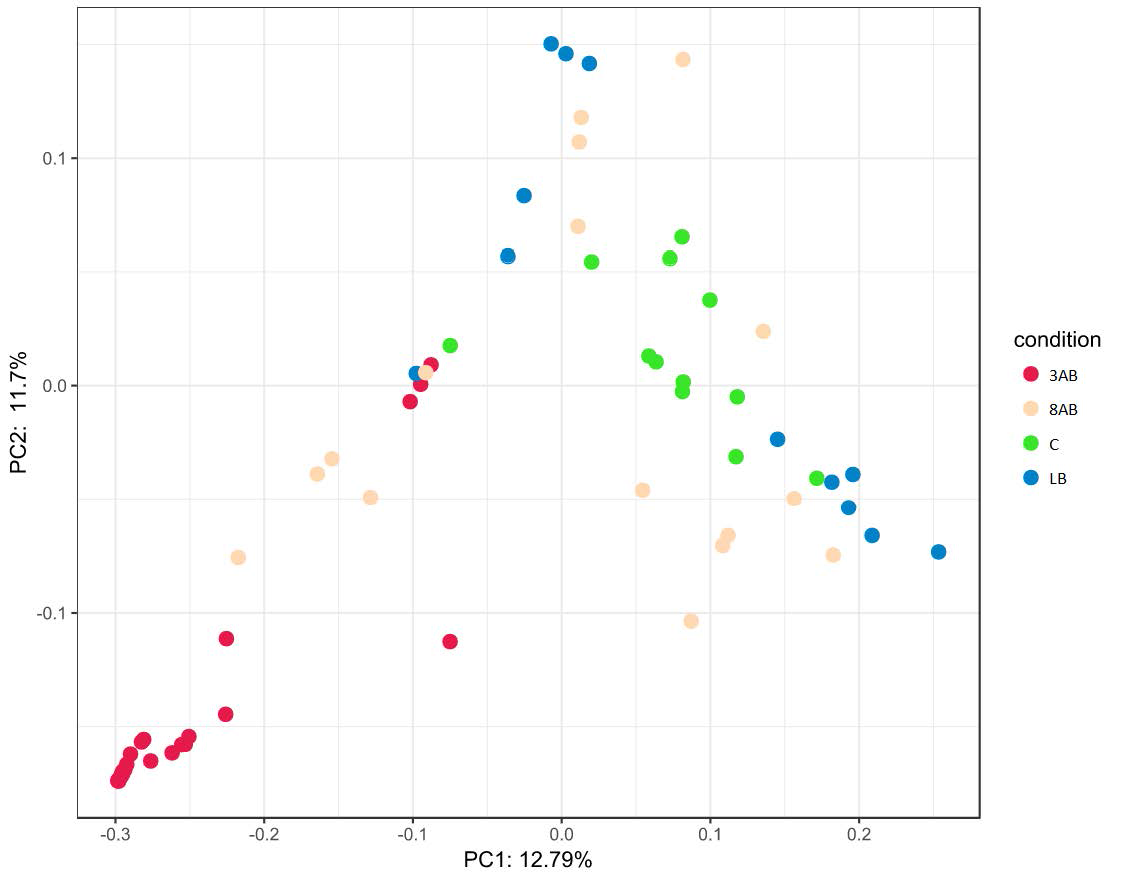
**

**B**

**
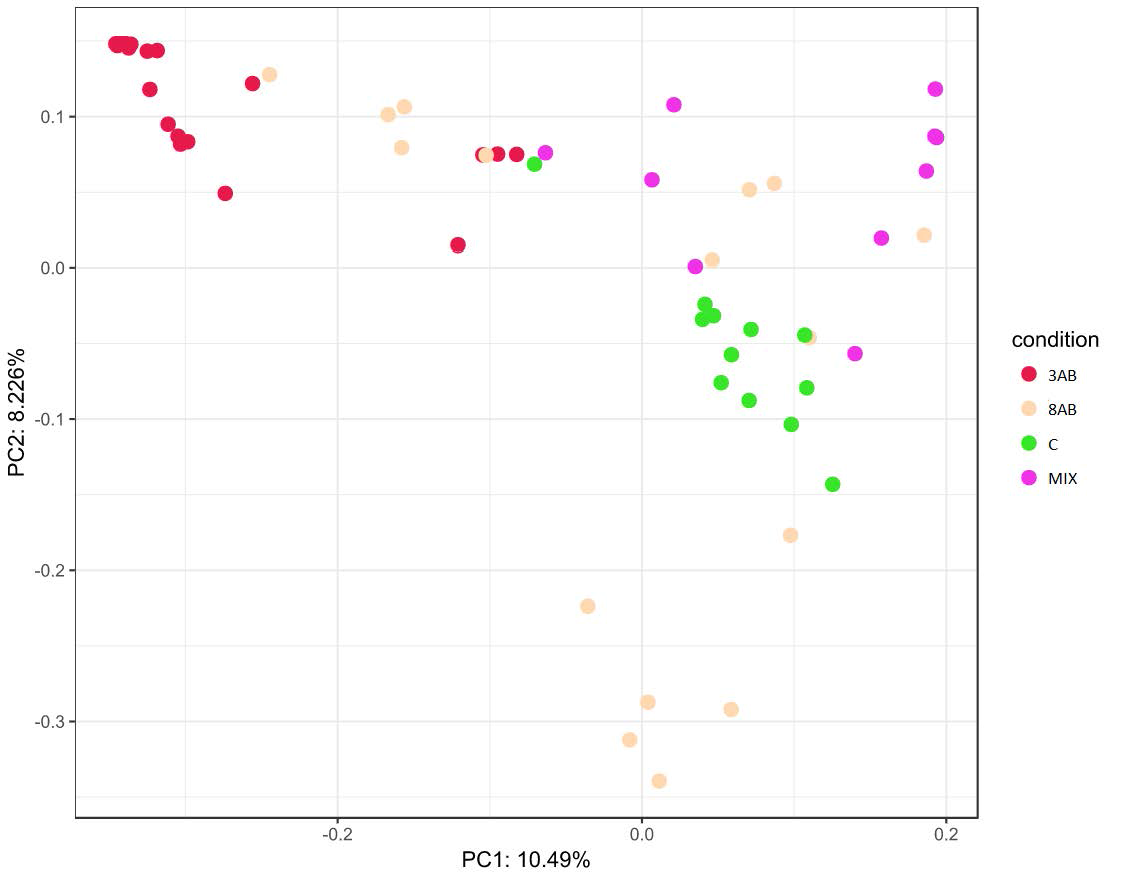
**

**C**

**
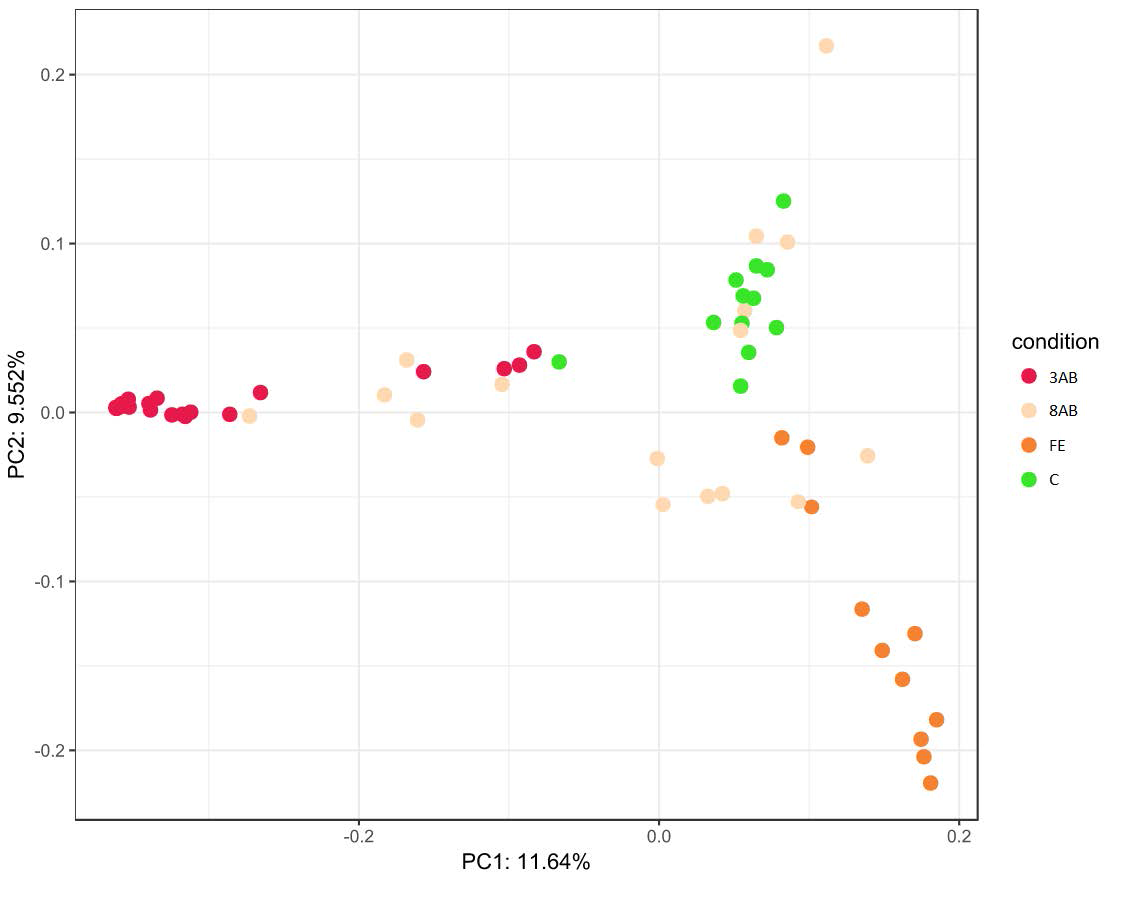
**

**D**

**
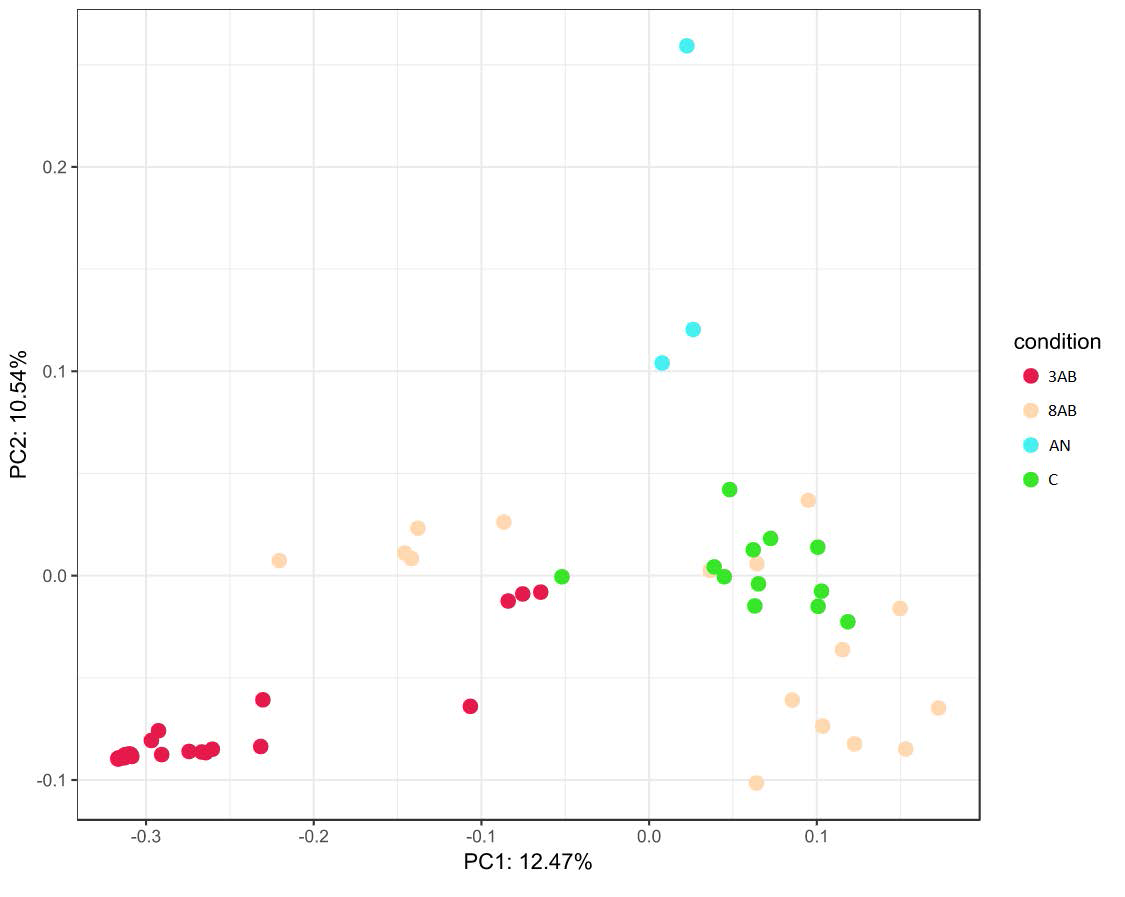
**

**Figure S4**

**A**

**
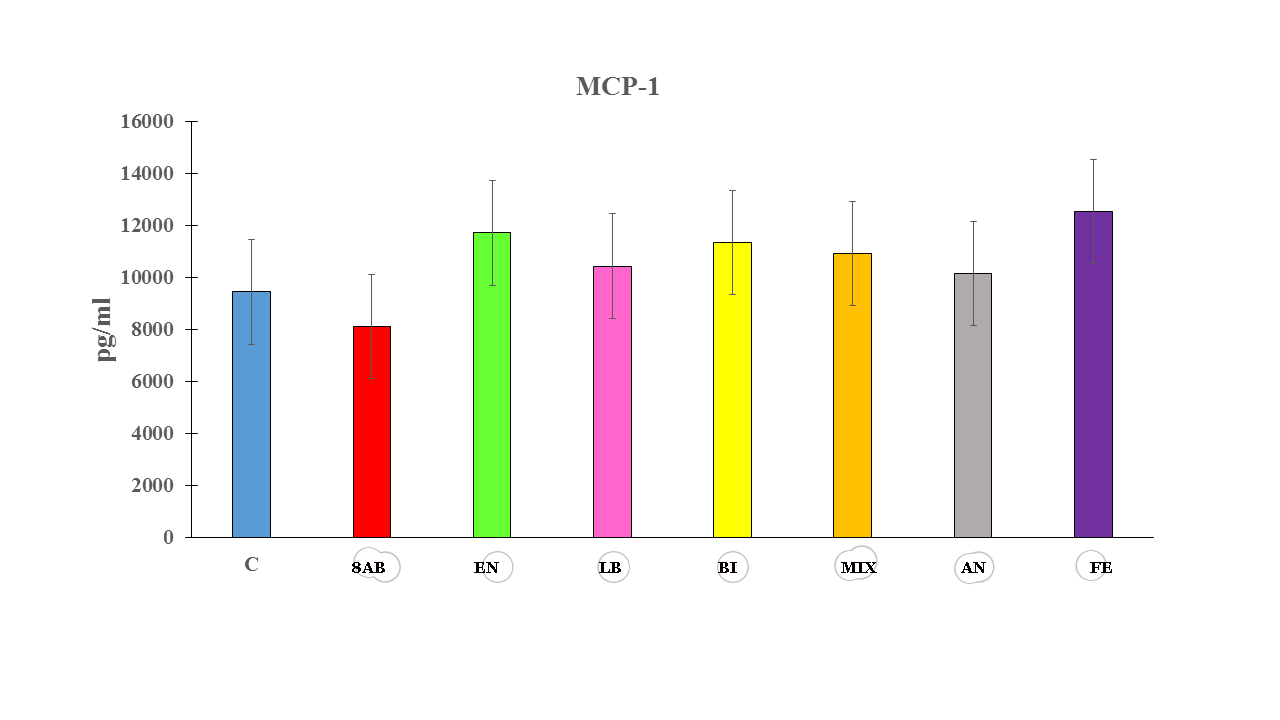
**

**B**

**
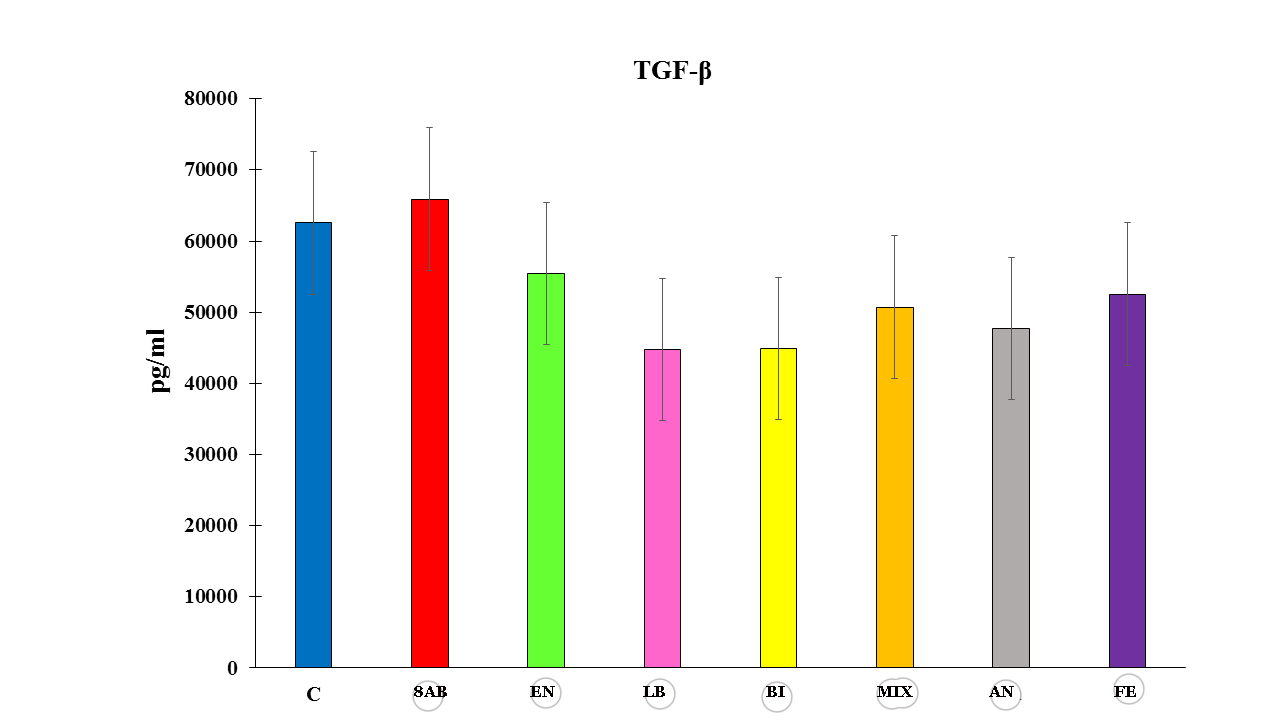
**

**Table S1**

DNA primers used in this study

| V3-V4 16S region sequencing primers | | Amplicon size bp |
| --- | --- | --- |
| Forward (341) | tcgtcggcagcgtcagatgtgtataagagacagcctacgggnggcwgcag | 464 |
| Reverse (785) | gtctcgtgggctcggagatgtgtataagagacaggactachvgggtatctaatcc |  |

**Species identification primers**

| Species | Forward | Reverse | Amplicon size bp |
| --- | --- | --- | --- |
| *E. faecium* | ttgaggcagaccagattgacg | tatgacagcgactccgattcc | 658 |
| *Lactobacillus spp.* | tcggctatcact tctggatgga | ccattgtggaag attccctactgc | 166 |
| *Bifidobacterium spp.* | gcgtgcttaacacatgcaagtc | cacccgtttccaggagctatt | 123 |

**Primers for the determination of the virulence genes of Enterococcus**

| Gene | Forward | Reverse | Amplicon size bp |
| --- | --- | --- | --- |
| *gelE* | accccgtatcattggttt | acgcattgcttttccatc | 419 |
| *esp* | ttgcataatgctagtccacgacc | gcgtcaatcggaagaatcat | 933 |
| *sprE* | gcgtcaatcggaagaatcat | cggggaaaaagctacatcaa | 233 |
| *fsrB* | tttattggtatgcgccacaa | tcatcagaccttggatgacg | 316 |
| *asa1* | ccagccaactatggcggaatc | cctgtcgcaagatcgactgta | 529 |
| *cylA* | actcggggattgataggc | gctgctaaagctgcgctt | 688 |
| *cylB* | attcctacctatgttctgtta | aataaactcttcttttccaac | 542 |
| *cylM* | gattggaatgtgggaatcctaa | acttccggcaacctttagtgta | 825 |
| *efaA* | cgttagctgcttgcgggaatc | ccatactacgtttatcgacac | 735 |
| *acm* | aagcacattcgatggtttttg | ggaactggttcccaattcttt | 423 |
| *TnVan1546 (vanA)* | ttcatgttccacgaaccagag | cgttgaacgaacgattgaaaa | 540 |
| *agg* | aagaaaaagaagtagaccaac | aaacggcaagacaagtaaata | 1552 |

**Table S2**

Results of comparison of intestinal microbiota composition in rats from experimental and control groups by metagenome analysis (Genus level)

| Genus/groups | 3AB | 8AB | AN | FE | EN | LB | BI | MIX |
| --- | --- | --- | --- | --- | --- | --- | --- | --- |
| *Bacteroides* | ↓ p=0.0001 | ↑ p=0.043 | ↑ p=0.0045 | ↑ p=0.0049 | ↑ p=0.0048 |  |  | ↑ p=0.0052 |
| *Bifidobacterium* |  | ↑ p=0.00172 | ↑ p=0.0045 |  |  |  | ↑ p=0.0047 |  |
| *Clostridium* | ↓ p=0.0001 |  |  |  |  | ↓ p=0.024 |  |  |
| *Enterobacter* |  |  |  |  |  |  |  |  |
| *Enterococcus* | ↑ p=0.0054 | ↑ p=0.001 |  |  |  |  |  |  |
| *Faecalibacterium* | ↓ p=0.042 |  |  |  |  | ↑ p=0.0050 | ↑ p=0.0046 |  |
| *Klebsiella spp.* | ↑ p=0.0001 | ↑ p=0.0032 | ↑ p=0.0045 |  | ↑ p=0.0048 | ↑ p=0.0051 |  | ↑ p=0.0052 |
| *Lactobacillus spp.* | ↓ p=0.0001 | ↓ p=0.002 |  | ↓ p=0.022 | ↓ p=0.021 |  |  | ↓ p=0.026 |
| *Oscillospira spp.* | ↓ p=0.0001 | ↓ p=0.033 | ↓ p=0.019 | ↓ p=0.023 | ↓ p=0.022 |  |  |  |
| *Parabacteroides* | ↓ p=0.0009 |  |  | ↓ p=0.019 | ↓ p=0.018 | ↑ p=0.0048 |  |  |
| *Paraprevotella spp.* | ↓ p=0.0035 |  |  |  | ↓ p=0.019 |  |  | ↓ p=0.026 |
| *Prevotella spp.* | ↓ p=0.0001 | ↓ p=0.0004 | ↓ p=0.019 | ↓ p=0.023 | ↓ p=0.022 |  | ↓ p=0.021 |  |
| *Proteus spp.* | ↑ p=0.0062 | ↑ p=0.0001 | ↑ p=0.0045 |  | ↑ p=0.0048 |  |  | ↑ p=0.0048 |

**Table S3**

Results of comparison of intestinal microbiota composition in rats from experimental and the control groups by metagenome analysis (Family level)

| Family/groups | **C** | **8AB** | **AN** | **BI** | **EN** | **FE** | **LB** | **MIX** |
| --- | --- | --- | --- | --- | --- | --- | --- | --- |
| *Alcaligenaceae* |  | ↑ p=0.0013 | ↑ p=0.0001 | ↑ p=0.0002 | ↑ p=0.00011 | ↑ p=0.0004 | ↑ p=0.0086 | ↑ p=0.0001 |
| *Bacteroidaceae* | ↑ p=0.002 |  | ↑ p=0.0003 |  | ↑ p=0.0002 | ↑ p=0.0003 |  |  |
| *Bifidobacteriaceae* |  | ↑ p=0.0019 | ↑ p=0.00020 | ↑ p=0.0005 | ↑ p=0.0179 | ↑ p=0.047 | ↑ p=0.082 | ↑ p=0.0036 |
| *Enterobacteriaceae* | ↓ p=0.0001 | ↑ p=0.0005 | ↑ p=0.033 | ↑ p=0.043 | ↑ p=0.0044 |  | ↑ p=0.0094 | ↑ p=0.0014 |
| *Enterococcaceae* | ↓  p =0.0001 | ↑ p=0.0006 |  | ↑ p=0.0178 |  |  |  |  |
| *Erysipelotrichaceae* | ↑ p=0.002 |  |  |  | ↑ p=0.0203 | ↑ p=0.0204 |  |  |
| *Helicobacteraceae* | ↑ p=0.001 |  |  |  | ↓ p=0.049 | ↓ p=0.042 |  |  |
| *Lachnospiraceae* | ↑ p=0.0001 |  | ↓ p=0.025 | ↑ p=0.001 | ↑ p=0.0002 |  |  |  |
| *Lactobacillaceae* | ↑ p=0.0022 |  |  |  |  | ↓  p= 0.0202 |  |  |
| *Pasteurellaceae* |  | ↑ p=0.0008 | ↑ p=0.0101 | ↑ p=0.036 | ↑ p=0.013 | ↑ p=0.014 | ↑ p=0.082 | ↑ p=0.036 |
| *Peptostrepto-coccaceae* | ↑ p=0.0008 | ↑ p=0.0001 | ↑ p=0.0002 | ↑ p=0.0002 | ↑ p=0.0003 |  |  | ↑ p=0.0006 |
| *Porphyromonadaceae* | ↑ p=0.0001 | ↓ p=0.002 | ↓ p=0.0031 |  | ↓ p=0.00012 |  |  |  |
| *Prevotellaceae* | ↓ p=0.0001 | ↓ p=0.0004 | ↓ p=0.0005 | ↓ p=0.0003 | ↓ p=0.0006 | ↓ p=0.0008 |  | ↓ p=0.0009 |
| *Ruminococcaceae* | ↑ p=0.0001 | ↓ p=0.0024 |  |  | ↓ p=0.0005 | ↓ p=0.0006 |  | ↓ p=0.043 |
| *S24* | ↑ p=0.0001 | ↓ p=0.0128 | ↓ p=0.025 |  | ↓ p=0.0102 |  |  |  |
| *Sphingomonada-ceae* |  |  |  |  |  |  | ↑ p=0.0129 |  |
| *Staphylococcaceae* | ↑ p=0.0015 |  |  |  |  |  |  |  |
| Veillonellaceae | ↑ p=0.0003 | ↓ p=0.003 | ↓ p=0.03 |  |  |  | ↓ p=0.04 |  |
|  |  |  |  |  |  |  |  |  |
